# Self-reported actual and desired proportion of sitting, standing, walking and physically demanding tasks of office employees in the workplace setting: do they fit together?

**DOI:** 10.1186/s13104-017-2829-9

**Published:** 2017-11-17

**Authors:** Birgit Wallmann-Sperlich, Josephine Y. Chau, Ingo Froboese

**Affiliations:** 1 Institute for Sports Science, Julius-Maximilians University Würzburg, Am Judenbühlweg 11, Würzburg, 97082 Germany; 2 Prevention Research Collaboration, School of Public Health, The University of Sydney, Charles Perkins Centre, Sydney, Australia; 3 Institute of Health Promotion and Clinical Movement Science, German Sport University Cologne, Cologne, Germany

**Keywords:** Office-workers, Physical activity, Sitting time, Cross-sectional

## Abstract

**Objective:**

Occupational sitting time in white-collar workers represents a prominent contributor to overall daily sitting time, which is associated with various health risks. Workplace interventions intending to reduce sitting time during work typically focus on replacing sitting with standing. The aim was to investigate and compare actual and desired proportions of time spent sitting, standing, walking, and doing physically demanding tasks at work reported by desk-based workers. Cross-sectional data were collected from German desk-based workers (n = 614; 53.3% men; 40.9 ± 13.5 years). All were interviewed about their self-reported actual and desired level of sitting, standing, walking and physically demanding tasks at work.

**Results:**

Desk-based workers reported to sit 73.0%, stand 10.2%, walk 12.9% and do physically demanding tasks 3.9% of their working hours. However, the individuals desire to sit, stand, walk and do physically demand tasks significantly different [53.8% sit, 15.8% stand, 22.8% walk, physically demanding tasks (7.7%), p < 0.001]. The present data revealed greatest mismatch between the desk-based workers’ actual and desired time for sitting and walking. Health promotion programs should offer not only options for more standing but also opportunities for more walking within the workplace setting to better match workers’ desires.

## Introduction

Research shows that high levels of sedentary behaviour are associated with negative health outcomes and all-cause mortality [1, 2]. Occupational sitting time is considered to be a prominent contributor to overall daily sitting time in white-collar workers [3–5] being particularly exposed to the health risks of prolonged sitting [6–8]. Consequently, reducing sitting time during working hours has been highlighted in the past few years by health promotion efforts in the workplace setting of office workers [9, 10]. Interventions that aim to reduce sitting time during work [11–13] often substitute sitting with standing, i.e. through combined sit and stand desks [14–16]. However, these interventions were developed without taking the office workers preference into account. To the best of our knowledge, there is little research about how long desk-based workers desire to sit, stand, walk or perform physical demanding tasks at their workplace. Knowledge about the workers’ desire in this regard could be helpful (i) to initiate countermeasures against prolonged and uninterrupted sitting in the workplace setting, and (ii) to stimulate greater compliance among desk-based office workers to promote health.

Hence, the aim of this study was to examine and compare self-reported actual and desired amount of work time spent sitting, standing, walking and doing physically demanding tasks in a desk-based workforce of German citizens. Data of this study were analysed previously to identify sociodemographic, health-related, and psycho-social variables of workday sitting including having a height-adjustable desk [17].

## Main text

### Methods

#### Study design

In spring 2016 we conducted a nationwide cross-sectional questionnaire-based telephone study on health behaviours including questions about self-reported sitting time and physical activity (PA) in the workplace setting in Germany. Survey methods have been described before [17]. Pre-tests were conducted in February 2016 for face validity and participant comprehension of the questionnaire with n = 9 participants and the designated professional interviewers were trained in administering the computer-assisted standardised questionnaire. Pre-tests revealed no changes necessary for the selected questions. All study procedures were approved by the Ethics Committee of the German Sport University in Cologne.

#### Sample

In total, 2830 representative residents (1386 men, 1444 women) from all 16 German federal states who were over 18 years of age (mean 50.4 ± 18.3 years) were interviewed. The sample was taken from the ADM Pool for Telephone Samples as described in more detail in [17]. The response rate for the study sample was 13.5%. In this study, we only included participants (i) who were working including participants in trainings and education, (ii) who specified that their work is a predominantly desk-based job and (iii) who answered all questions regarding actual and desired proportion of sitting, standing, walking and doing physically demanding tasks. Because of these inclusion criteria and our data-cleaning process, we excluded data from respondents not working (n = 1202), not working predominantly desk-based jobs (n = 868) and because of missing values in one or all questions (n = 146). Ultimately, our sample consisted of 614 participants (53.3% men; 40.9 ± 13.5 years).

#### Measures

##### Self-reported actual sitting time and PA in the workplace setting

The Occupational Sitting and Physical Activity Questionnaire (OSPAQ) was used to assess self-reported PA and sitting time in the office environment [18]. The OSPAQ is a validated instrument asking the participant to indicate the proportion of work time that she or he spends sitting, standing, walking, and doing physical demanding tasks on a typical workday in the last 7 days as well as the number of hours they had worked in the last 7 days (weekly working hours) and the number of days they were at work. To calculate the minutes per workday participants spent sitting, standing, walking, and doing physically demanding tasks, self-reported percentage time spent in each activity was multiplied by the number of hours worked/day at work. The OSPAQ shows excellent test–retest reliability (ICC from 0.73 to 0.90) and moderate validity for estimating time spent sitting and standing at work compared to accelerometers (r = 0.65 and r = 0.49) [18, 19].

##### Desired sitting time and PA in the workplace setting

The desired sitting time and PA in the workplace setting was assessed similar to the proportion question of the OSPAQ with the following introduction phrase: “If you have the choice, what proportion of work time would you like to spend sitting, standing, walking, and doing physically demanding tasks on a typical workday?” To calculate the desired minutes per workday participants want to spend sitting, standing, walking, and doing physically demanding tasks, self-reported desired percentage time spent in each activity was multiplied by the number of hours worked/day at work. These items were developed specifically for this study and pre-tested for face validity and participant comprehension.

##### Socio-demographic variables

The demographic variables included self-reported age and gender. Additional socio-demographic variables comprised education and income levels. Education was categorised into the following levels based on the German school system: ‘no school graduation’, ‘10 years of education’, ‘12 years of education’, ‘13 years of education’ and ‘first university degree or higher’. Household net income per month was assessed in nine categories and summarised in three groups based on tertiles: ‘low income’ (< 1500€), ‘middle income’ (1500€–2499€), and ‘high income’ (€ > 2500€).

#### Data analysis

We employed the data processing software PASW© (Version 23) for all statistical analyses. To calculate the difference between the actual proportions and minutes per working day of sitting, standing, walking and doing physically demanding tasks at work and the desired proportions and minutes per working day we applied the Wilcoxon signed-rank test for the overall sample as well as for men and women separately. Multiple linear regression analyses investigated associations of socio-demographic correlates and the dependent variables of “difference of actual-desired minutes of sitting”, “difference of actual-desired minutes of standing”, “difference of actual-desired minutes of walking” and “difference of actual-desired minutes of doing physically demanding tasks”. We selected the forced entry method to explore the associations. Socio-demographic variables comprised age (continuous variable), education (five categories), income level (three categories) and working hours/working day (continuous variable). Statistical significance was set at a level of p < .05.

### Results

Distribution of the proportion and minutes/working day of sitting, standing, walking and doing physically demanding tasks were not normally distributed. The participants reported to sit 73.0 ± 21.7%, stand 10.2 ± 12.4%, walk 12.9 ± 10.9% and doing physically demanding tasks 3.9 ± 8.2% of their working day. The participants desired to sit 53.8 ± 23.6% (< .001), stand 15.8 ± 13.7% (< .001), walk 22.8 ± 17.5% (< .001) and do physically demanding tasks 7.7 ± 12.7% (< .001) of their working day (see Table 1).

**Table 1:** Results of Wilcoxon signed-rank test for differences in the self-reported actual and the desired proportion and minutes per workday of sitting, standing, walking and physically demanding tasks in desk-based workers

	All (n = 614)		Men (n = 327)		Women (n = 287)	
	Actual	Desired	Actual	Desired	Actual	Desired
Proportion of workday sitting in % (SD)	73.0 (21.7)	53.8 (23.6)***	73.6 (20.1)	55.3 (21.6)***	72.4 (23.5)	52.0 (25.6)***
Proportion of workday standing in % (SD)	10.2 (12.4)	15.8 (13.7)***	10.4 (11.8)	15.7 (12.2)***	10.0 (13.1)	15.9 (25.6) ***
Proportion of workday walking in % (SD)	12.9 (10.9)	22.8 (17.5)***	12.5 (9.9)	21.9 (16.4)***	13.2 (11.9)	23.8 (18.6)***
Proportion of workday physically demanding tasks in % (SD)	3.9 (8.2)	7.7 (12.7)***	3.5 (7.6)	7.1 (10.4)***	4.4 (8.9)	8.4 (14.9)***
Min/workday of sitting (SD)	321.5 (152.5)	238.1 (139.7)***	339.1 (146.3)	255.4 (133.1)***	301.5 (157.1)	218.4 (144.5)***
Min/workday of standing (SD)	44.0 (56.6)	69.5 (68.4)***	47.7 (55.7)	74.7 (66.9)***	39.7 (57.5)	63.4 (69.8)***
Min/workday of walking (SD)	55.0 (51.8)	96.9 (85.5)***	58.1 (52.2)	101.2 (86.7)***	51.4 (51.3)	92.1 (83.9)***
Min/workday of physically demanding tasks (SD)	17.0 (37.5)	33.0 (59.5)***	17.5 (41.3)	31.1 (46.8)***	16.5 (32.6)	35.2 (71.3)***

*** p < 0.001

Regression models explained 4% for the dependant variable “difference sitting” and “difference standing” and less than 1% for “difference walking” and “difference doing physically demanding tasks” (see Table 2). The first model revealed a positive association (β = .20) between the “hours/workday” and the dependant variable “difference sitting”, meaning that the more hours/day the participant spends working the greater the difference between “actual minus desired sitting time” is, which implicates that the longer the workdays the less the workers want to spend sitting. In the second model “hours per workday” were negatively associated (β = − .20) with the dependant variable “difference standing”, demonstrating that the longer the workday the smaller the difference is between “actual minus desired standing time”, meaning the longer the wish is to stand during working hours.

**Table 2:** Results from multiple linear regressions on contribution of socio-demographic correlates and daily working hours on the dependent variables

	Difference sitting (n = 422)			Difference standing (n = 422)			Difference walking (n = 422)			Difference physically demanding tasks (n = 422)		
	B	SE B	β	B	SE B	β	B	SE B	β	B	SE B	β
Gender	− 5.64	10.59	− .03	1.06	6.04	.01	− .80	7.07	− .01	5.47	6.16	.05
Age	− .06	.45	− .01	− .25	.26	− .05	.44	.30	.07	− .12	.26	− .02
Education	7.10	5.59	.06	− 3.93	3.19	− .06	− .36	3.74	− .01	− 2.72	3.26	− .04
Income	− 3.46	8.05	− .02	− .433	4.59	− .01	4.52	5.38	.04	− .74	4.68	− .01
Hours per workday	8.39	2.06	.20***	− 4.77	1.18	*−* .20***	− 2.52	1.38	− .09	− 1.17	1.20	− .05
	*Adj. R* ^*2*^ = .036			*Adj. R* ^*2*^ = .039			*Adj. R* ^*2*^ = .002			*Adj. R* ^*2*^ = − .004		

*B* unstandardized beta; *SE* *B* standard error of beta; *β* standardized beta; *** p < 0.001

Dependent variables: “difference self-reported actual - desired min/working day of sitting”, “difference self-reported actual - desired min/working day of standing”, “difference self-reported actual - desired min/working day of walking”, and “difference self-reported actual - desired min/working day of physically demanding tasks”

### Discussion

The novel finding of this study is that desk-based workers desire to sit for approximately half (4.0 h) of their working day, which differs considerably from their self-reported actual sitting proportion of over 70% (5.4 h). Interestingly the desired amount of standing and walking time (about 2.7 h) in our sample mirrors nicely a recently released expert statement. This report was conducted from a health perspective without looking at preferences and recommends desk-based workers to accumulate 2 h of standing and light PA during working hours and progress to a total of 4 h/day (50% of an 8-h work day) [20]. Our results lend some support to the recommended reduction of sitting time to only 50% in the workplace setting which seems feasible in light of workers’ relatively congruent preferences for sitting, standing and walking. Alternatively, these results reflect respondents’ awareness of recent guidance about occupational sitting time. The implementation of workers’ personal preferences for sitting and PA could make a substantial change and be important to reduce the risk for various negative health outcomes [6, 7]. However, it should be noted that intervention studies have not been able to achieve this level of sitting reduction [21]. Regarding reducing occupational sitting time, positive attitude [22, 23], social norms, behavioural control and self-regulatory-skills can be important components in behaviour modification [23–25]. Habit also explains sedentary behaviour in the occupational setting [26]. Consequently, interventions need to pursue habit breaking attempts, e.g. through environmental modifications [26–28], sit-stand desks [14–16], active design building [29], or behaviour change strategies [23, 25, 28], as well as supportive workplace policies for more PA [9].

The second main finding of this study is that desk-based workers expressed desire to walk significantly more during working hours than to stand and even desired more physically demanding tasks. The desired amount of walking is about 46 min/8 h-workday more than the self-reported amount, while the standing difference is only about 26 min/8 h-workday more. To date most of the health promotion activities in the desk-based working environment that are waged under the key message “sit less, move more” [9, 30–32] achieves a reduction of sitting time through i.e. active workstations, but without increasing stepping [12, 15, 33] or vigorous PA. Our results suggest that future interventions in this area should focus more on increasing walking and PA during working hours. This is also supported by the greater improvement of cardio-metabolic risk factors through walking or light, moderate and vigorous PA [34]. Options that increase walking and PA during working hours are warranted and should be included as part of a range of strategies in workplace programs that aim to reduce sitting and increase PA.

Overall, the present data suggest a minor role of socio-demographic factors such as gender, age, education or income on the dependent variables and recommend actions to reduce workplace sitting and increase PA for all workers, especially for workers with long working hours. However, we did not assess pre-existing health problems which could influence desired occupational sitting and PA and should be considered in future studies.

### Conclusion

The results of this study suggest that health promotion activities to reduce sitting time in the workplace setting are supported by the desire of the desk-based workers, which is a good foundation for workplace wellness actions. Our findings suggest health promotion programs should offer not only options for more standing but also opportunities for more walking within the workplace setting to better match workers’ desires.

## Limitations

The strength of the present study is the big nation-wide sample rather than the analysis of one specific company setting. From this point of view, the results provide a representative insight of the desired sitting and PA of desk-based workers in Germany. However, the low response rate is a limitation, which potentially may have been a result of the overall mean duration of this telephone-based health survey (approx. 22.5 min). Comparing our study to other surveys [35], the present response rate seems acceptable. A further limitation is that this study obtained data based on self-reported sitting and PA in the workplace setting. Self-reporting of sitting is prone to potential bias via misclassifications or social desirability and could have been controlled through objective measures [36], but this was logistically not feasible in this survey. Nevertheless, the specified 73.0% of sitting during working hours of the workers in the present study may be underestimated, but not differing much from the measured 78.8–82% of sitting during working hours in Australia [3, 12].

## Abbreviations

OSPAQ: occupational sitting and physical activity questionnaire
PA: physical activity
